# Pycnodysostosis: An Anaesthetic Approach to This Rare Genetic Disorder

**DOI:** 10.1155/2013/716756

**Published:** 2013-03-14

**Authors:** Rajeev Puri, Arpita Saxena, Awak Mittal, Zia Arshad, Yogita Dwivedi, Trilok Chand, Apurva Mittal, Archna Agrawal, Jay Prakash, Sathiyanarayanan Pilendran

**Affiliations:** ^1^Department of Anaesthesia and Critical Care, S. N. Medical College, Agra, India; ^2^Department of Orthopaedics, S. N. Medical College, Agra, India

## Abstract

Pycnodysostosis (the Toulouse-Lautrec syndrome) is a rare autosomal-recessive disorder of osteoclast dysfunction. This disorder was first described by Maroteaux and Lamy in 1962. We describe anaesthetic management of a 35-year-old female having pyknodysostosis with fracture shaft left femur with anticipated difficult intubation. Therefore, spinal anesthesia was planned for her fracture fixation. The intra- and postoperative period remains uneventful.

## 1. Introduction

Pycodysostosis is a rare autosomal-recessive disorder of osteoclast dysfunction due to mutation of cathepsin K gene [1] causing osteosclerosis. The disease shows equal sex distribution with high parental consanguinity, having an incidence of 1.7 per 1 million births [2, 3]. This disorder is characterized by short stature, increased bone density, short and stubby fingers, fragile bones that may fracture easily, and craniofacial abnormalities caused by delayed suture closure. Patients usually present with frequent fractures even after minor trauma.

## 2. Case Report

**Figure 1:**
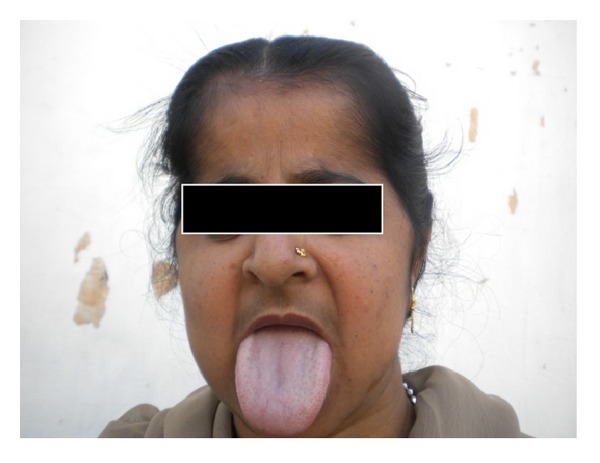
Patient with large protruding tongue.

**Figure 2:**
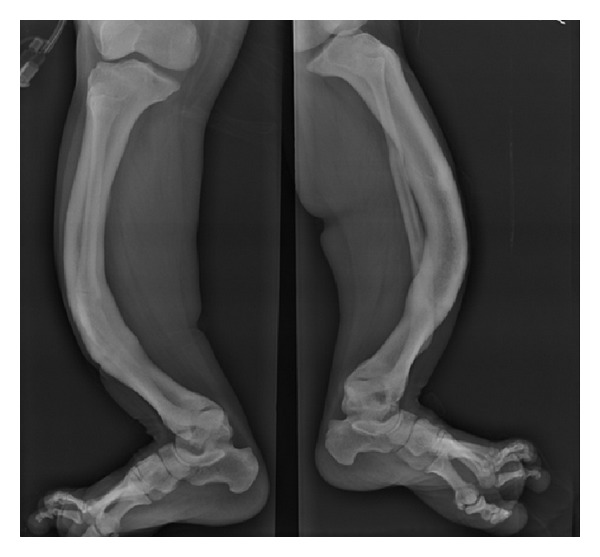
X-ray showing bowing of long bones.

A 34-year-old, 45 kg patient having Pycodysostosis was planned for elective femur plating under spinal anesthesia. The patient had past history of spontaneous fractures which were managed conservatively. Her mental and sexual developments were normal. Patient had large protruding tongue (Figure 1) and Mallampati grade IV with a history of snoring. Patient had characteristic short stature, particularly limbs, short broad hands, frontal and occipital bossing, and chest deformities. Radiograph demonstrates increased bone density with hypoplastic clavicle, narrow intervertebral spaces, and bowing long bones (Figure 2). The patient height was 130 cm, upper limb to lower limb ratio was 60/76, and arm span was 128 cm. Patient had no known history of allergy to any drug. Laboratory investigations including serum electrolytes, ECG, and X-ray of chest were within normal limits.

Inside the operation theatre intravenous access was secured with an 18-gauge cannula, and preloading was done with 500 mL of Ringer's lactate. Urinary catheterization was done. Monitoring of electrocardiograph, heart rate, SpO2, and NIBP was done. Patient preoperative vitals were within normal limits. Ceftriaxone 1 gm was administered. Under strict aseptic conditions, spinal anesthesia was given at lumber space L2-3 with 26-gauge spinal needle in sitting position. Drug used was 2.5 mL of bupivacaine heavy (0.5%). The patient then positioned supine, and a sensory level up to T12 was achieved. 1 mL of midazolam was given to the patient to allay operative anxiety. Total duration of surgery was 80 minutes. Intraoperative and postoperative period was uneventful.

## 3. Discussion

**Figure 3:**
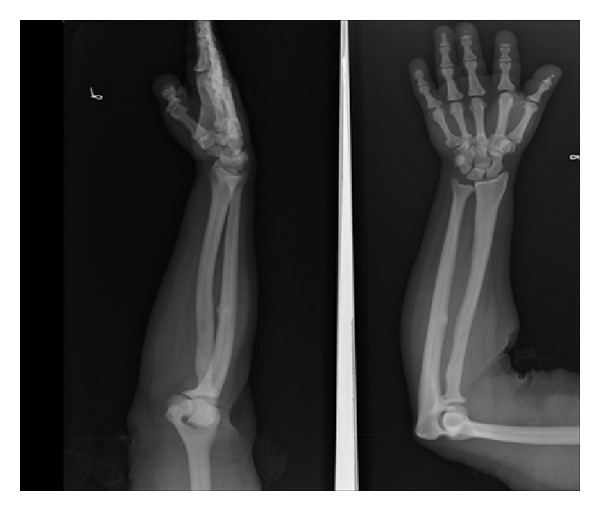
X-ray showing sclerosis of terminal phalanges.

Pycodysostosis is a rare autosomal-recessive disorder of osteoclast dysfunction [4], and the understanding of early and delayed radio-clinical manifestations of pycnodysostosis is very important since it resembles cleidocranial dysostosis and osteopetrosis [5]. The features which differentiates it are short stature, brachycephaly, generalized diffuse osteosclerosis, sclerosis of the terminal phalanges (Figure 3), hypoplastic clavicles, and history of multiple fractures of long bones [6]. The jaw and collar bone (clavicles) are also particularly prone to fractures.

Craniofacial features include a large head with front parietal bossing, open soft cranial sutures and fontanelles, depressed nasal bridge, a high arched grooved palate, maxillary hypoplasia, mandibular fractures, osteomyelitis, malpositioned teeth, elongated soft palate precipitating mouth breathing, and heavy snoring in addition to periapical cementoma-like lesions in the mandible [7, 8]. Many of these findings were present in our case which differentiates her from osteopetrosis and cleidocranial dysostosis. In osteopetrosis, there is no delayed closure of cranial sutures, no phalangeal, or clavicle hypoplasia. Cleidocranial dysostosis is transmitted by autosomal dominant inheritance, open fontanels and cranial sutures are also observed at an advanced age, and there is no phalangeal or clavicle hypoplasia.

Pycodysostosis has been described all over the world with minimal difference affecting all races regardless of sex and age; the youngest patient reported was a nine-month-old baby and the oldest was 45 years in age [2]. PKD in children is commoner in males than in females, occurring at a ratio of 2 : 1 [9]. The sclerosing activity of Pycodysostosis is due to a genetic defect located on chromosome 1q21. This anomaly consists of mutations that produce changes in a lysosomal cystine protease, cathepsin K [4], the expression of which is reduced in the osteoclasts of these patients [10]. Patient's one elder brother also had similar features.

Various reports have been published regarding Pycodysostosis which presents clinical and radiological features which differentiates it from others, but little or none have been talked about giving anaesthesia in these patients. We were dealing with a very rare case with anticipated difficult intubation due to high arched palate, mandibular hypoplasia, and large protruding tongue, which made it Mallampati grade IV. Also sniffing position and airway manipulations during endotracheal intubation may predispose patient to trauma and fracture. So general anaesthesia was avoided, and spinal anaesthesia was chosen for femoral plating. History of obstructive sleep apnea may have a risk of airway obstruction in postoperative period; hence, it should be carefully addressed.

The diagnosis of Pycodysostosis is primarily based on clinical features and radiographs. In lack of any specific treatment of Pycodysostosis, which is mainly supportive, our main aim is to prevent minor trauma, which may cause iatrogenic fracture in the patient. Such precautions include careful handling of an affected patient, with minimal manipulations that are safe and do not require too much impact.

In conclusion, undoubtedly these patients pose challenge to anaesthesiologists, but proper preoperative patient assessment, planning, and multidisciplinary approach are the keys to successful outcome in these patients.
